# Differential proteome analysis in adolescent idiopathic scoliosis patients with thoracolumbar/lumbar curvatures

**DOI:** 10.1186/s12891-019-2640-y

**Published:** 2019-05-24

**Authors:** Hiroto Makino, Shoji Seki, Isao Kitajima, Hiraku Motomura, Makiko Nogami, Yasuhito Yahara, Naoko Ejiri, Tomoatsu Kimura

**Affiliations:** 1 Department of Orthopaedic Surgery, University of Toyama, Faculty of Medicine, 2630 Sugitani, Toyama, Toyama 930-0194 Japan; 2 Department of Clinical Laboratory and Molecular Pathology, University of Toyama, Toyama, Japan

**Keywords:** Adolescent idiopathic scoliosis, Vitamin D binding protein, Two-dimensional fluorescence difference gel electrophoresis, Thoracolumbar curvature

## Abstract

**Background:**

Although the pathogenesis of adolescent idiopathic scoliosis (AIS) remains unclear, there are little evidences of the pathogenesis in patients with thoracolumbar/lumbar AIS. The purpose of this study was to identify proteins or proteomes that may be causally related to the pathogenesis of AIS with structured thoracolumbar/lumbar curvature using two-dimensional fluorescence difference gel electrophoresis (2D-DIGE).

**Methods:**

A total of 20 control volunteers and 61 AIS in patients with thoracolumbar/lumbar curvature were included. First, the plasma samples of each five AIS with pure thoracolumbar/lumbar curvature and control samples were subjected to 2D-DIGE analysis. Protein spots that were expressed differently by the AIS and control groups were selected and identified by nanoscale liquid chromatography–tandem mass spectrometry (nanoLC-MS/MS) analysis. To characterize the differently-expressed proteins in AIS patients, we performed functional pathway analysis using the Protein ANalysis THrough Evolutionary Relationships (PANTHER) system. Additionally, the proteins were compared between control and AIS using western blotting. Lastly, prospectively collected 15 control and 41 AIS with thoracolumbar/lumbar curvature samples were compared to the differentially expressed proteins.

**Results:**

A total of 3862 ± 137 spots were detected, of which 11 spots met the criteria when compared with controls. Nine proteins were identified by nanoLC-MS/MS. Functional analysis showed the association of the proteins in AIS patients with blood coagulation using the PANTHER system. Of the proteins, vitamin D binding protein (DBP) significantly correlated with Cobb angle in thoracolumbar/lumbar curvatures. DBP expression of the prospectively collected AIS samples were significantly higher than those of controls (*P* < 0.05).

**Conclusions:**

This study suggests that DBP and several coagulation-related proteins may play a role in the pathogenesis of AIS. DBP appears to be a marker of severity of AIS with thoracolumbar/lumbar curvature.

## Background

Adolescent idiopathic scoliosis (AIS) is a spine deformity that becomes noticeable during the pubertal growth spurt [1]. It is characterized by a 3-dimensional curvature in the absence of congenital vertebral malformation. AIS is found in approximately 0.5–4% of the population, including mild cases, but severe cases are rare [1]. Since the curvature in AIS sometimes progresses [2], and progression of the curvature may lead to respiratory dysfunction [3] and back pain [4], bracing of curvatures > 25° is usually undertaken [5]. Surgery is considered to be required for ≥40–50° of curvature [5] Bracing can have tolerability problems, and multilevel fusion surgery, although the reported complication rates may be acceptable [6], can be associated with cosmetic problems due to scarring and with serious complications such as neural impairment, vascular injury, and deep wound infection [6]. There are no prophylactic measures available. The pathogenesis of AIS is still unclear, although recent studies have suggested the role of genetic variants in bone mineralization pathways in AIS [7, 8]. However, these genetic variants have not been associated with clinical significance. Although, there have been a lot of reports in genome-wide association study of common diseases, the clinical applications have not been seen in most of the studies. Bone mineral density in AIS patients is decreased [9], as is the ability of marrow mesenchymal cells to undergo osteogenic differentiation [10]. One study reported that 65% of their AIS patients had osteopenia and 59% had high values for the bone resorption marker tartrate-resistant acid phosphatase serum band 5 (TRAP5b) [11]. Wang et al. revealed decreased levels of the osteoblast-associated transcription factor Runx2 mRNA in patients with AIS [12].

It is unknown whether all types of AIS are the result of the same pathogenesis. Lenke et al. classified six types of curvature for surgical treatment [13]. Although Lenke classification is generally used for the surgical treatment, it helps to know the origin of curvature. Several studies [7, 8, 10, 12] focused on patients with Lenke type 1 or 2 (pure structured thoracic curvature) which are common in their populations. Although the pathogenesis of AIS is generally considered to be multi-factorial in origin, possible susceptibilities to AIS in tissues relate to nerves, muscles, ligaments, discs, skeletal proportions and asymmetries [14], and subsequent relative anterior spinal overgrowth drives 3D scoliosis deformity. Considering pathogenesis of the scoliosis, the thoracic curvature is likely to be affected by the growth of ribs, or rib cages, or shallow chest with cardio-thoracic disproportion [15]. It seems that there are some pathological differences between thoracic and thoracolumbar/lumbar scoliosis. To the best of our knowledge, there have been little reports of the pathogenesis of AIS limited to thoracolumbar/lumbar curvature using differential proteome analysis.

First, to identify additional proteins or proteomes related to the pathogenesis of AIS, we characterized the proteomes associated with thoracolumbar/lumbar curvature using two-dimensional fluorescence difference gel electrophoresis (2D-DIGE). Then, we selected proteins of interest among those expressed in different amounts in normal and AIS plasma samples identified with 2D-DIGE analysis, and examined the relationship between the proteins’ levels and clinical factors in thoracolumbar/lumbar curvature. The purpose of this study was to identify proteins or proteomes associated with the pathogenesis of AIS with structured thoracolumbar/lumbar curvature using 2D-DIGE.

## Methods

### The aim, design, and setting of study

Prospectively collected blood samples of AIS with structured thoracolumbar/lumbar curvature were used and analyzed. This study was approved by the Ethics Committee of our institute, and written informed consent was obtained from all subjects and their parents before participating in this study.

### Characteristics of participants

Subjects were 61 AIS patients with thoracolumbar/lumbar curvatures scheduled for surgery and 20 volunteers for this case-control study. All patients had undergone physical and radiological examination such as x-ray, computed tomography, and magnetic resonance imaging to confirm the diagnosis of AIS. Control subjects were patients visiting our hospital for other reasons than AIS such as extremity fracture or postural abnormality without scoliosis with X-ray. The mean ± SD age was 13.9 ± 2.1 years in the AIS group, and 13.5 ± 0.9 years in the control group (Table 1). Blood samples were drawn from all subjects into a tube containing a proteinase inhibitor (BD™ P100; Becton, Dickinson and Company, San Jose CA USA) before surgery in the patients with AIS or at the time when control subjects were judged that original disease was stable, and the plasma fraction was isolated.

**Table 1:** The demographics of the subjects in this study

	Control	AIS patients with thoracolumbar/lumbar (Lenke type 3–6)	p
Number	20	61	
Age (years)	13.5 ± 0.9	13.9 ± 2.1	N.S.
Gender	boy; 2, girl; 19	boy; 2, girl; 59	N.S.

### 2D-DIGE protocol

We used the pooled plasma from the five patients with pure structured thoracolumbar/lumbar curvature (Lenke type 5) and from the five control subjects for 2D-DIGE analysis, as well as a sample of pooled plasma from both patients and controls. These three mixtures were subjected to protein removal (Multiple Affinity Removal Spin Cartridge®; Agilent Technologies, Palo Alto CA, USA), including albumin, IgG, IgA, transferrin, haptoglobin and antitrypsin and to centrifugal concentration (Amicon Ultla-4, 3 kDa; Merck Millipore, Darmstadt, Germany). The resulting samples were then labeled with the carbocyanine dyes Cy2, Cy3, and Cy5 (Table 2). We then performed two-dimensional electrophoresis under the following conditions three times by alternatively labeling the control and AIS samples: First-dimensional isoelectric focusing was done with 36 kVh (300 V, 5 h; 300–3500 V, 1.5 h; 3500 V, 10 h) using the Multiphore II flatbed electrophoresis system (GE Healthcare, Milwaukee WI, USA) with immobilized pH gradient strips (24 cm, pH 3-11NL, GE Healthcare) to which a total of 150 μg samples (50 μg each of AIS, control, and pooled samples) was applied. The strips were equilibrated in two steps. First, the strips were incubated with the equilibration solution (50 mM Tris, pH 8.8, 6 M urea, 30% glycerol, 2% SDS) including 0.25% w/v dithiothreitol (DTT) for 10 min at room temperature, followed by incubation with the above equilibration solution plus 4.5% w/v iodoacetamide for another 10 min at room temperature. After equilibration, sodium dodecyl sulfate-polyacrylamide gel electrophoresis (SDS-PAGE) was performed for 15 h at 3 W with an Ettan DALT II system (GE Healthcare) using 12% polyacrylamide gels. Subsequently, we scanned the gels with a Typhoon 9400 scanner (GE Healthcare). Differential analysis software (DeCyder v7.0®, GE Healthcare) was used to analyze the DIGE image. Protein spots that were under- or overexpressed in different volumes by the patients compared to controls with a spot volume ratio of > 2 or < 0.5 and *p* < 0.05 (Student’s *t*-test) were selected.

**Table 2:** Fluorescent dye assignment in two-dimensional fluorescence difference gel electrophoresis (2D-DIGE) analysis

Gel No.	Cy 2	Cy 3	Cy 5
1	Pool (standard)	Control	AIS
2	Pool (standard)	AIS	Control
3	Pool (standard)	Control	AIS

### Nanoscale liquid chromatography-tandem mass spectrometry (nanoLC-MS/MS)

Additional 500 μg plasma samples from Lenke 5 subjects and controls were run under the same conditions to obtain enough protein for mass spectrometry (MS) analysis. Following fixation and staining with a ruthenium-based protein gel stain (Sypro®Ruby; Bio-Rad Laboratories, Richmond CA, USA), the under- or overexpressed proteins that had been identified in 2D-DIGE analysis were excised from the Sypro®Ruby-stained gels by a robotic picker (Spot-picker**®**, GE Healthcare) and digested using 2 μL trypsin and 50 mM ammonium hydrogen carbonate overnight. After digestion, the tryptic peptides were centrifuged and dried, followed by dissolving in 20 μL 1% formic acid.

Nanoscale liquid chromatography–tandem mass spectrometry (nanoLC-MS/MS) analysis was performed with high-pressure liquid chromatography (UltiMate® 3000 HPLC; Dionex, Sunnyvale CA, USA) and mass spectrometry (Q-Exactive Plus®, Thermo Scientific, Bremen, Germany). We used a search engine (MASCOT®, Matrix Science, London, UK) with the SWISS-PROT and NCBInr protein sequence databases to identify the proteins.

#### Protein network and functional analysis

To characterize the proteins expressed in different amounts in AIS patients, we performed functional pathway analysis using the Protein ANalysis THrough Evolutionary Relationships (PANTHER) system, ver. 11.1 [16] and identified associated gene ontology terms, biological processes, molecular functions, and physiological pathways.

#### Western blot

The expression of target proteins by all Lenke types in plasma was measured by western blotting analysis. A 0.5% dilution of plasma from the 5 control subjects and 20 AIS patients with structured thoracolumbar/lumbar curvature was applied to gels with lithium dodecyl sulfate (LDS) sample buffer and a reducing agent. Each concentration of protein in plasma was same (3.1 g/dl), and equal volume of plasma was loaded for western blotting analysis. We then performed electrophoresis at 200 V for 30 min and transferred the gels onto polyvinylidene fluoride membranes. The membranes were incubated with anti-vitamin D binding protein antibody (1:1000; sc-32,899, Santa Cruz Biotechnology, USA) and dissolved in 5% skim milk following blocking with 5% skim milk for an hour at room temperature. Secondary antibodies conjugated with horseradish peroxidase were applied and blots were visualized by ECL Prime Western Blot Detecting Reagent (GE Healthcare, USA). Image J software (NIH, USA) was used to measure the signal intensity of the bands.

#### Enzyme-linked ImmunoSorbent assay (ELISA)

To confirm the difference between other control and AIS with thoracolumbar/lumbar curvature, plasma DBP was measured with Human Vitamin D Binding Protein Quantikine ELISA Kit (R&D Systems, Minneapolis, MN, USA) according to the manufacturer’s instructions. Plasma from controls (*n* = 15) and the patients with thoracolumbar/lumbar curvature (*n* = 41) were measured in duplicate and compared.

#### Statistical analysis

Data are shown as the mean and standard deviation. We used ANOVA followed by the Tukey-Kramer post-hoc test to compare multiple groups and Student’s *t*-test to select proteins expressed in different volumes, and *p* values < 0.05 were considered statistically significant. We calculated correlation coefficients between the expression of a target protein and each continuous variable such as age, body mass index (BMI), bone maturity (Risser grade), and Cobb angle in the patients with AIS. Moreover, stepwise multiple linear regression was used to predict the expression of a focused protein based on age, BMI, Risser grade, and Cobb angle.

## Results

**Fig. 1:**
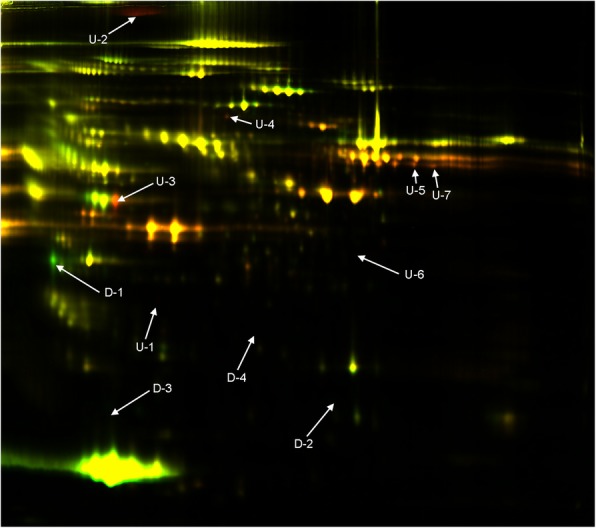
Representative 2D-DIGE overlays of plasma derived from Gel no. 1 [Cy3 (control), Cy5 (AIS), Cy2 (Pool)]. Spots for which the quantitative statistical analysis revealed more than 2.0-fold or less than 1/2 expression change and a Student’s *t*-test *p* < 0.05 in the AIS group are annotated by numbers. Upregulated spots are marked by “U,” and downregulated spots are marked by “D”

**Fig. 2:**
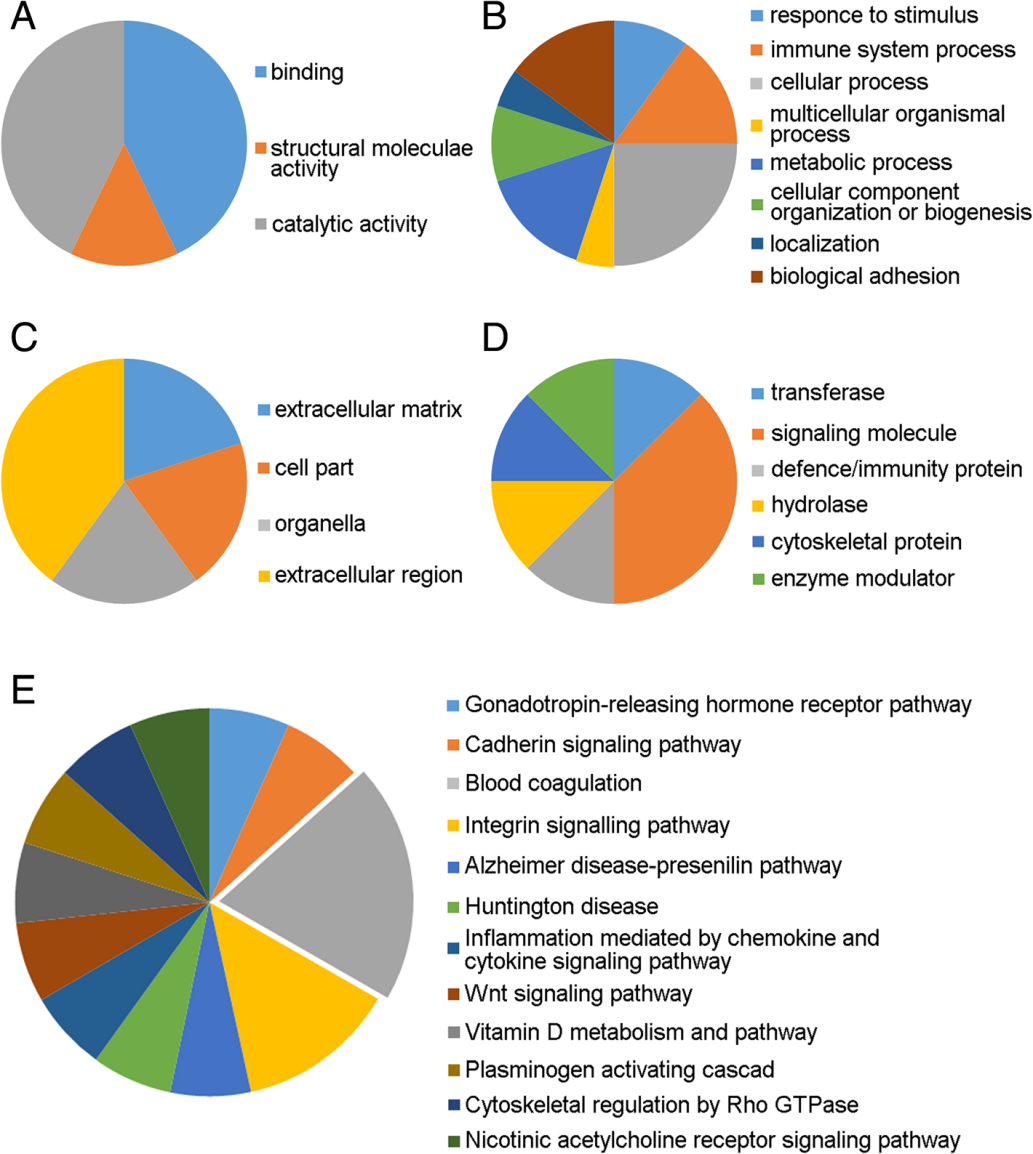
Functional clustering and pathways associated with the nine under- or overexpressed proteins identified in the AIS patients by PANTHER analysis. The pie charts represent molecular function (**a**), biological process (**b**), cellular component (**c**), protein class (**d**) and PANTHER pathway (**e**). All nine proteins are significantly associated with the blood coagulation pathway (*p* = 0.000148)

**Fig. 3:**
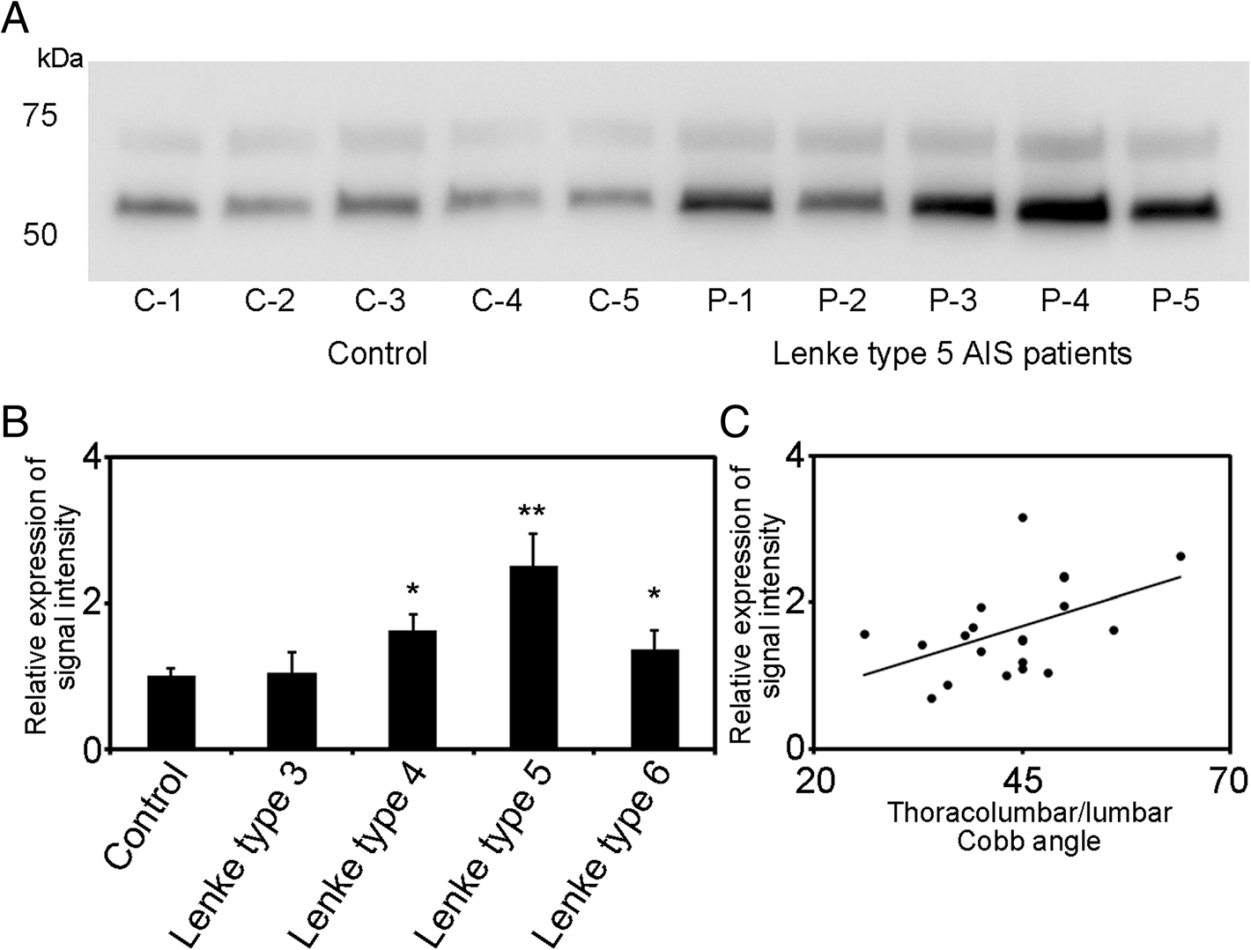
We validated the expression of vitamin D binding protein (DBP) in plasma. **a** Western blotting in controls and Lenke type 5 patients. **b** Density analysis of the patients with AIS (Lenke type 3–6; *n* = 20) plasma vs. control (*n* = 5). The expression of DBP in plasma was significantly increased in the patients with operatively treated Lenke 4, 5 and 6 AIS compared to control subjects (** *p* < 0.01, * p < 0.05). **c** This shows a significant correlation between DBP expression and Cobb angles in thoracolumbar/lumbar curvatures (r = 0.43, *p* = 0.03)

**Fig. 4:**
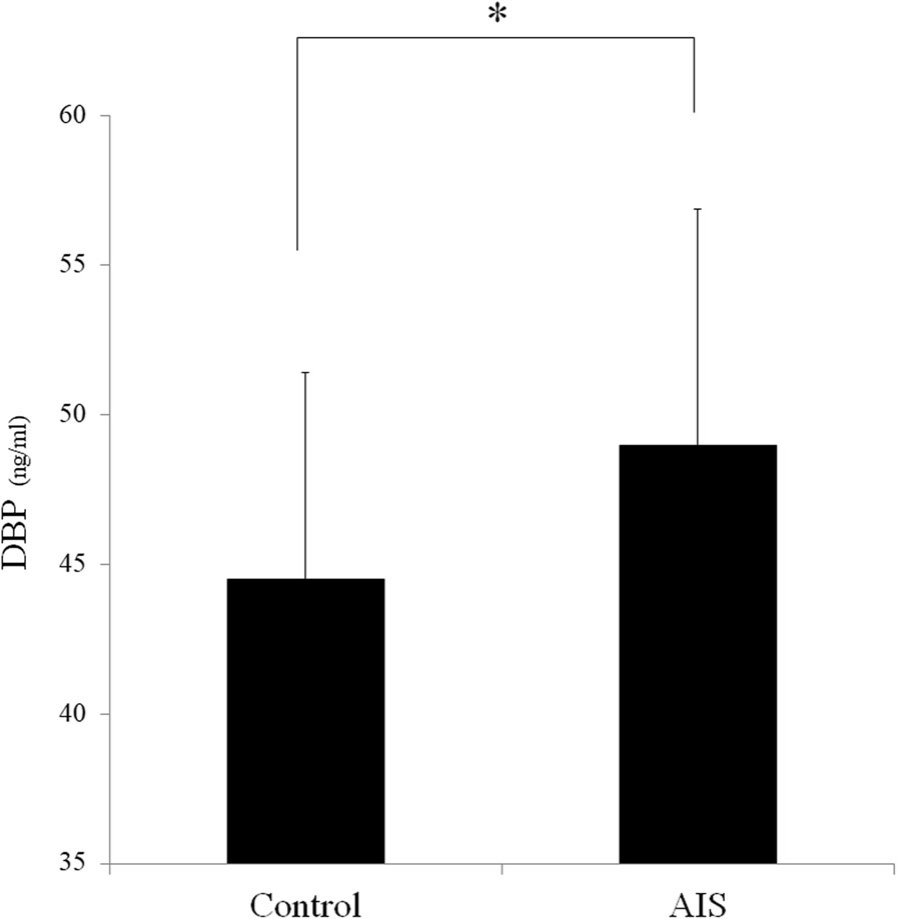
Expression analysis of vitamin D binding protein (DBP) in plasma was performed by ELISA. There was significant difference between AIS (*n* = 41) with thoracolumbar/lumbar curvature and control (*n* = 15) subjects (* *p* < 0.05)

A total of 3862 ± 137 spots were detected on three independent gels. Among these protein spots, 11 spots met the two criteria of expression change of > 2 × or < 0.5 × the volume compared to the control group and a significant Student’s *t*-test result (*p* < 0.05). The 11 spots with different volumes of expression were dissected and identified in LC-MS/MS analysis (Fig. 1). Nine proteins were identified from the 11 spots by nanoLC-MS/MS (Table 3). Among these, actin (cytoplasmic1), fibronectin, vitamin D binding protein (DBP), coagulation factor XIII A chain, fibrinogen alpha chain, and complement factor H-related protein 1 were increased and complement C3, adiponectin, and prothrombin were decreased in AIS patients with Lenke type 5 curvature compared to controls. Functional analysis using the PANTHER system revealed the association of over- and underexpressed proteins in AIS patients with gene ontology (GO) terms including three molecular functions (binding; 42.9%, catalytic activity; 42.9%, structural molecule activity; 14.3%, Fig. 2a), eight biological processes (cellular process; 25%, immune system process; 15%, metabolic process; 15%, biological adhesion; 15%, response to stimulus; 10%, cellular component organization or biogenesis; 10%, multicellular organismal process; 5%, localization; 5%, Fig. 2b), four cellular components (extracellular region; 40%, extracellular matrix; 20%, cell part; 20%, organelle; 20%, Fig. 2c) and six protein classes (signaling molecule; 37.5%, transferase; 12.5%, defense/immunity protein; 12.5%, hydrolase; 12.5%, cytoskeletal protein; 12.5%, enzyme modulator; 12.5%, Fig. 2d). Additionally, PANTHER pathway analysis showed the association of the nine over- or underexpressed proteins in AIS patients with blood coagulation (*p* = 0.000148, Fig. 2e). Of the nine over- or underexpressed proteins in AIS patients, we focused on DBP and did the following analysis because we felt it was the most significant protein and is closely related to bone metabolism. Expression of DBP in plasma was significantly increased in Lenke 5 patients at 2.5 × the control values (Fig. 3a, b). Additionally, the expression of DBP in plasma was significantly increased in AIS patients with operatively treated Lenke type 3–6 curvatures compared to controls. There was no difference in the expression of DBP between controls and AIS patients with Lenke type 3 curvatures. Among the three curve types, the expression of DBP was highest in the Lenke 5 patients (Fig. 3b). Plasma DBP levels were not significantly correlated with age or bone maturity (Table 4). They did correlate significantly with BMI and Cobb angle in main thoracic and thoracolumbar/lumbar curvatures (Fig. 3c, Table 4). In a stepwise multiple regression analysis, the Cobb angle of thoracolumbar/lumbar curvatures and BMI were selected as independent variables (R^2^ = 0.36, *p* < 0.01, Table 5). Furthermore, we analyzed the difference of DBP expression between prospectively collected AIS samples with thoracolumbar/lumbar curvature and controls. DBP expression in AIS was significantly higher than that of control (*p* < 0.05, Fig. 4).

**Table 3:** Identification of under- and overexpressed proteins by nanoLC-MS/MS

Spot No.	protein	NW^a^	PI^b^	Ratio^c^	*p*-value^d^	Score^e^	Peptide^f^	Coverage^g^
U-1	Actin, cytoplasmic 1	42,052	5.29	4.98	0.00044	338	7	35%
U-2	Fibronectin	266,052	5.46	4.55	0.011	1640	25	22%
U-3	Vitamin D-binding protein	54,526	5.40	2.40	0.000033	8417	26	72%
U-4	Coagulation factor XIII A chain	83,728	5.75	2.33	0.00013	728	14	29%
U-5	Fibrinogen alpha chain	95,656	5.70	2.08	0.00059	17,627	26	44%
U-6	Complement factor H-related protein 1	38,766	7.38	2.06	0.00059	725	6	18%
U-7	Fibrinogen alpha chain	95,656	5.70	2.04	0.0005	10,673	25	42%
D-1	Complement C3	188,569	6.02	−3.19	0.0043	2819	16	21%
D-2	Complement C3	188,569	6.02	−2.84	0.0076	437	7	13%
D-3	Adiponectin	26,511	5.42	−2.50	0.021	59	1	0.17%
D-4	Prothrombin	42,108	5.31	−2.06	0.022	92	4	5%

a; Relative molecular mass, b; Isoelectric point, c; Mean volume ratio of AIS group vs. control group (AIS/Control, −X = 1/X), d; Student’s t-test, e; Based on the MOWSE logarithmic scoring algorithm; a score of ≥ 64 indicates significance, f; Number of peptide fragments matching the candidate protein, g; Percentage of sequences hit for registered full sequence

**Table 4:** Correlation between DBP in plasma and clinical factors

	AIS (Lenke type 3–6)	Correlation coefficient	*P* value
Age (y. o.)	14.9 ± 2.4	0.02	0.93
Body mass index (kg/ m^2^)	19.7 ± 2.5	0.45	0.04
Risser grade	3.5 ± 1.2	0.20	0.40
Main thoracic Cobb (°)	44.7 ± 13.0	−0.31	0.10
Thoracolumbar/ lumbar Cobb (°)	43.6 ± 8.5	0.43	0.03

**Table 5:** Multiple linear regression analysis for variables predicting plasma DBP

	B^1^	SE B^2^	β^3^	p	95% confidence interval
Body mass index	0.11	0.05	0.45	0.03	0.02, 0.21
Thoracolumbar/lumbar Cobb angle	0.035	0.01	0.47	0.02	0.01, 0.06

R2 = 0.36 (0.43), F (2, 17) = 6.4, *p* = 0.009, LOF = 0.13 (*p* = 0.99). B^1^; Estimated partial regression coefficient, SEB^2^; standard error of estimated partial regression coefficient, β^3^; tandardized partial regression coefficient

## Discussions

We identified nine proteins over- or underexpressed by AIS Lenke type 5 patients compared to control subjects using 2D-DIGE. Subsequently, we characterized those proteins with GO terms using the PANTHER system and revealed an association with the blood coagulation pathway. We were also interested in DBP, and examined the relationship between the expression of DBP in plasma and clinical factors of the patients with Lenke type 3–6 including type 5 as owing structured thoracolumbar/lumbar curvature, finding a significant correlation with Cobb angle. There haven’t been any reports on the correlation between AIS and upregulation of plasma levels of DBP. The novelty of our results was that DBP in plasma was increased in AIS patients with pure structured thoracolumbar/lumbar curvature.

Most of the proteins over- or underexpressed by AIS Lenke type 5 patients are involved in the coagulation pathway. Supporting this result, there have been some studies reporting blood coagulation abnormalities in the patients with AIS [17, 18]. Ryan KM et al. showed that children with scoliosis have a higher prevalence of preoperative coagulation abnormalities compared to normal healthy volunteers [17]. Ho WK et al. also reported a high prevalence of coagulation abnormalities among patients with AIS [18]. Bosch P et al. recently reported a case of massive bleeding during posterior fusion surgery despite no coagulation abnormalities [19]. They considered that upregulation of fibrinolysis is involved, but it might be considered that there are blood coagulation abnormalities specific to AIS that are undetectable on routine coagulation studies.

We particularly focused on DBP among the nine proteins over- or underexpressed by AIS Lenke type 5 patients. The reason why we selected it was that low bone mineral density in AIS patients has been reported in previous studies [11], implicating a bone metabolism disturbance in AIS pathogenesis. DBP, also known as GC (Group-specific component) globulin, is the major carrier protein of vitamin D as well as a scavenger of extracellular actin [20]. Some studies have reported DBP variants related to osteoporosis [21, 22]. Moreover, Wang et al. reported that vitamin D receptor and DBP genetic polymorphism correlated with susceptibility to AIS and efficacy of brace treatment [22]. Some studies showing the association between vitamin D and AIS have been reported. Goździalska et al. showed that there was a significantly lower level of 25-OH-D3 in girls with AIS than in healthy girls [23]. Balioglu et al. also reported that vitamin D levels were lower in patients with AIS [24]. It is still unknown how DBP protein levels affect vitamin D metabolism. As an index of vitamin D status, 25 (OH) D is measured in clinical practice, 85% of which is tightly bound to DBP and 10–15% loosely bound to albumin. The remaining small amount exists as free [25]. Bioavailable 25 (OH) D is believed to be free [26]. Increased DBP in the blood is thought to reduce free 25 (OH) D [27], and the association of free 25 (OH) D concentration with various diseases has been suggested [28, 29]. Together with the results of this study, vitamin D status seems to be a pivotal feature in AIS, and there is a possibility that DBP may be involved in the pathogenesis or severity of AIS.

DBP has a direct effect on bone metabolism. Its precursor, Gc protein-derived macrophage activating factor (Gc-MAF), activates osteoclasts [27]. Given the report that 65% of AIS patients had osteopenia and 59% of them had high values for TRAP5b [11], together with our results, increased plasma DBP in the patients with AIS might affect osteoclast activity and cause an imbalance between bone formation and resorption. We also found that DBP in plasma correlated positively with Cobb angle in thoracolumbar/lumbar curvatures in the patients with Lenke types 3–6 of AIS. Furthermore, multivariate analysis including other factors revealed that Cobb angle in thoracolumbar/lumbar curvatures correlated positively with DBP levels. These finding suggested that the levels of DBP might be able to predict a scoliotic curve progression. Additionally, multivariate analysis revealed that body mass index in AIS patients correlated positively with DBP levels. However, there is no significant difference between body mass index and Cobb angle (data not shown). There have been some reports of negative association between the DBP levels and body mass index [30]. It had thought that AIS patients were significantly underweight compared to healthy control [29]. Based on our data, the underweight in patients with AIS might be associated with the low DBP levels. A number of prospective analyses between the DBP levels in patients with AIS and their body mass index are desired in the future.

We performed 2D-DIGE using plasma from patients with Lenke type 5 AIS in this study. Previous studies on the pathogenesis of AIS mostly targeted all types of AIS, meaning that it included patients with Lenke types 1 and 2, which are thoracic curvatures. Because the thoracic scoliosis is affected by the growth of ribs or rib cages [14, 15], we have thought that there are some pathological differences between thoracic and thoracolumbar/lumbar scoliosis. Therefore, we performed 2D-DIGE using the plasma from the patients with pure thoracolumbar/lumbar curvature, Lenke type 5 AIS. This was aimed at obtaining new findings by targeting a more homogeneous population.

This study had several limitations. First, it was a single time-point analysis, and it is necessary to analyze how DBP changes with the process of disease severity over time. Second, the type of Lenke classification may change over time in each case. For example, it is necessary to examine how DBP levels change when, for example, Lenke type 5 becomes Lenke type 6. However, if the patients have originally thoracolumbar/lumbar curvature, their curvature will develop to Lenke type 3–6.

In conclusion, this study identified nine proteins over- or underexpressed by AIS Lenke type 5 patients compared to control subjects using 2D-DIGE. Most of these proteins were involved in the blood coagulation pathway. In addition, plasma DBP levels positively and significantly correlated to Cobb angle. Given that DBP is associated with bone metabolism, it might therefore play an important role in the pathogenesis of AIS or disease severity.

## Abbreviations

2D-DIGE: Two-dimensional fluorescence difference gel electrophoresis
AIS: Adolescent idiopathic scoliosis
BMI: Body mass index
DBP: Vitamin D binding protein
GO: Gene ontology
nanoLC-MS/MS: Nanoscale liquid chromatography–tandem mass spectrometry
PANTHER: Protein ANalysis THrough Evolutionary Relationships
TRAP5b: Tartrate-resistant acid phosphatase serum band 5
